# Brachial metastatic plexopathy as the inaugural manifestation of lung cancer: multimodality imaging

**DOI:** 10.1259/bjrcr.20150410

**Published:** 2016-11-02

**Authors:** Bruno Coulier, Oswald Van Cutsem, Patrick Mailleux, Fabienne Richelle

**Affiliations:** ^1^Department of Diagnostic Radiology, Clinique St Luc, Namur, Belgium; ^2^Department of Pneumology, Clinique St Luc, Namur, Belgium; ^3^Department of Nuclear Medicine, Clinique St Luc, Namur, Belgium

## Abstract

Metastatic infiltration of a peripheral plexus, also named metastatic plexopathy (MP), often results in severe pain and muscular weakness. This rather rare event may have a dramatic impact on the quality of life of patients affected by cancer. We hereby report a rare case of painful MP of the left cervicobrachial plexus presenting as the inaugural manifestation of poorly differentiated large-cell lung carcinoma in a 53-year-old patient. This responsible lung carcinoma was fortuitously diagnosed during MRI of the brachial plexus (BP). Complementary cancer staging was completed by contrast-enhanced multidetector CT, 18-fludeoxyglucose–positron emission tomography/CT and colour Doppler ultrasound of the BP. Although MRI remains the gold standard method for imaging the BP, our reported case emphasizes the alternative diagnostic capabilities of contrast-enhanced multidetector CT and ultrasound and confirms the high specificity of 18-fludeoxyglucose–positron emission tomography/CT in distinguishing brachial MP from secondary radiation plexopathy.

## Summary

We report a rare case of painful metastatic plexopathy (MP) of the left brachial plexus (BP) presenting as the inaugural manifestation of poorly differentiated large-cell lung carcinoma in a 53-year-old patient. This responsible lung carcinoma was fortuitously diagnosed during MRI of the BP. Complementary cancer staging was completed by contrast-enhanced multidetetor CT (MDCT), 18-fludeoxyglucose–positron emission tomography/CT (^18^F-FDG PET/CT) and colour Doppler ultrasound of the BP. Although MRI remains the gold standard method for imaging the BP, this case emphasizes the alternative diagnostic capabilities of contrast-enhanced MDCT and ultrasound and confirms the high specificity of ^18^F-FDG PET/CT in distinguishing brachial MP from secondary radiation plexopathy.

## Background

Metastatic infiltration of a peripheral plexus, also named MP, often results in severe pain and muscular weakness. This, fortunately, rather rare event may have a dramatic impact on the quality of life of affected cancer patients.^1^

The prevalence MP has retrospectively been estimated to be approximately 0.43% for the BP and 0.71% for the lumbosacral plexus. Nevertheless the exact prevalence of MP remains unknown because in many cases, the rather unspecific symptoms are attributed to other complications such as bony metastases.^1^ Thus, the diagnosis of MP remains a challenge and may be delayed.

## Clinical presentation

A 53-year-old patient presented to the orthopaedic department with a 5-month history of increasing and intractable left cervicobrachial pain. The pain had first been attributed to cervical osteoarthritis and/or rotator cuff tendinopathy. Shoulder CT arthrography had been performed but revealed normal findings except for an unusual evolving dystrophy of the humeral head. Previous CT of the cervical spine had also diagnosed significant multilevel cervical spinal stenosis, which was maximal at the level of C6–C7. During the past 2 months, the patient had developed monoplegia and muscular testing worsened.

## Investigations/imaging findings

**Figure 1.**
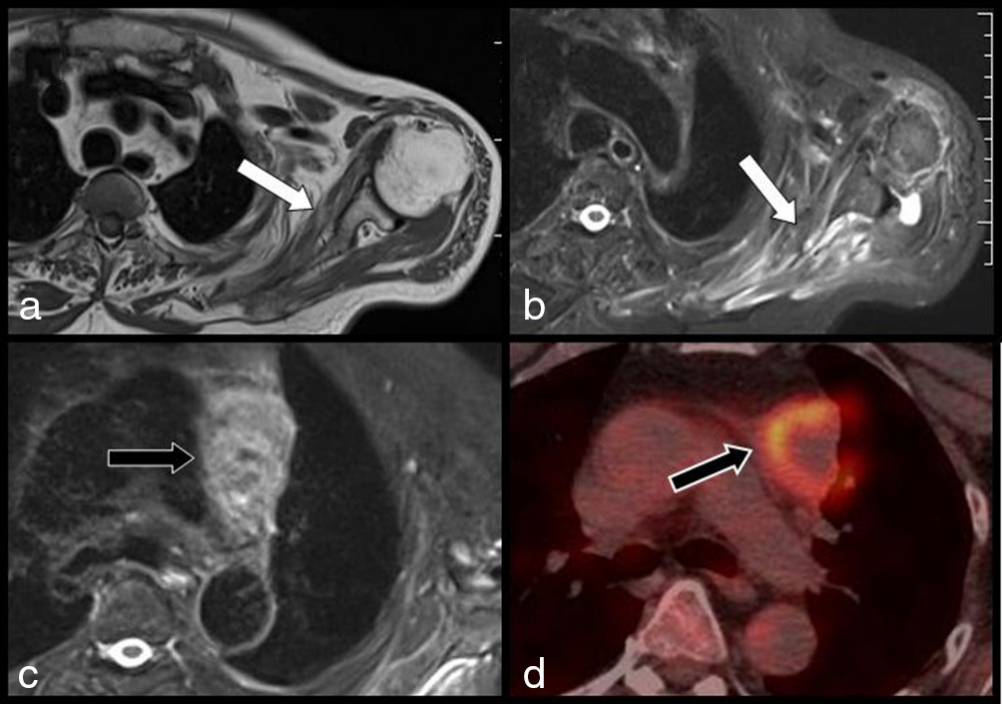
MRI views (a, b) of the left scapular area. *T*_1_ weighted image (a) showing diffuse atrophy of the scapular muscles (white arrow) and (b) typical denervation oedema (white arrow) is seen on *T*_2_ weighted image. *T*_2_ weighted image obtained more distally (c) in the mediastinum reveals a tumoral mass (black arrow) in the inferolateral portion of the anterior mediastinum. Corresponding 18-fludeoxyglucose–positron emission tomography/CT image (d) confirms the hypermetabolic tumoral mass (black arrow).

**Figure 2.**
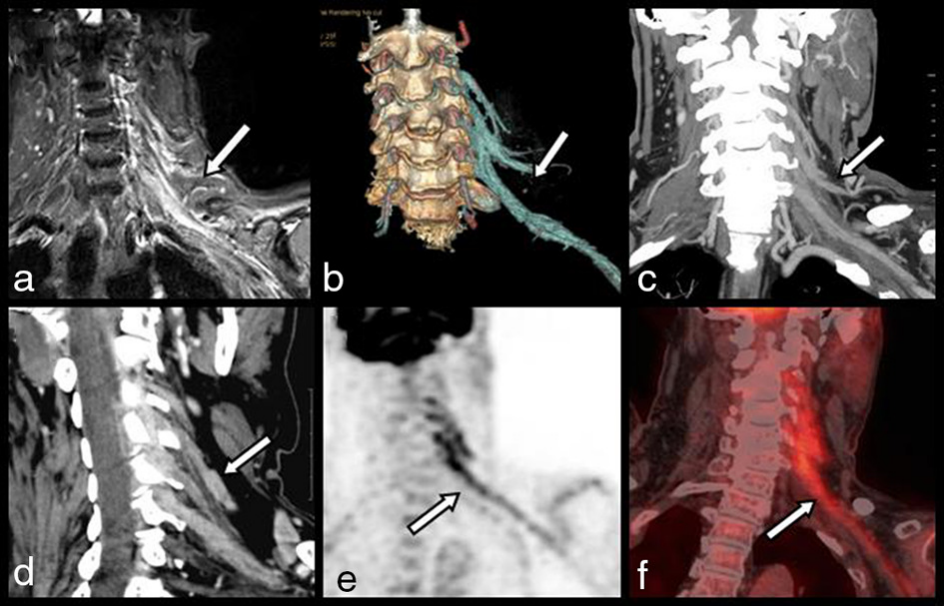
This series (a–f) compares the images of the brachial metastatic plexopathy (white arrows) obtained with the different imaging modalities. All images are coronal or coronal oblique views. (a) Coronal *T*_2_ weighted fat-saturated MRI illustrating the diffuse thickening with high signal intensity of the brachial plexus roots extending from the cervical spine to the axilla. Corresponding views of contrast-enhanced multidetector CT comprising: (b) selective volume rendering, (c) coronal maximal intensity projection and (d) coronal oblique multiplanar view. The images show diffuse thickening with massive contrast enhancement of the third, fourth and fifth and especially of the sixth left cervicobrachial roots extending from the neurovascular foramina to the axilla. Corresponding 18-fludeoxyglucose–positron emission tomography (e) and fused 18-fludeoxyglucose–positron emission tomography/CT (f) images clearly illustrate the high fludeoxyglucose uptake of the metastatic roots.

**Figure 3.**
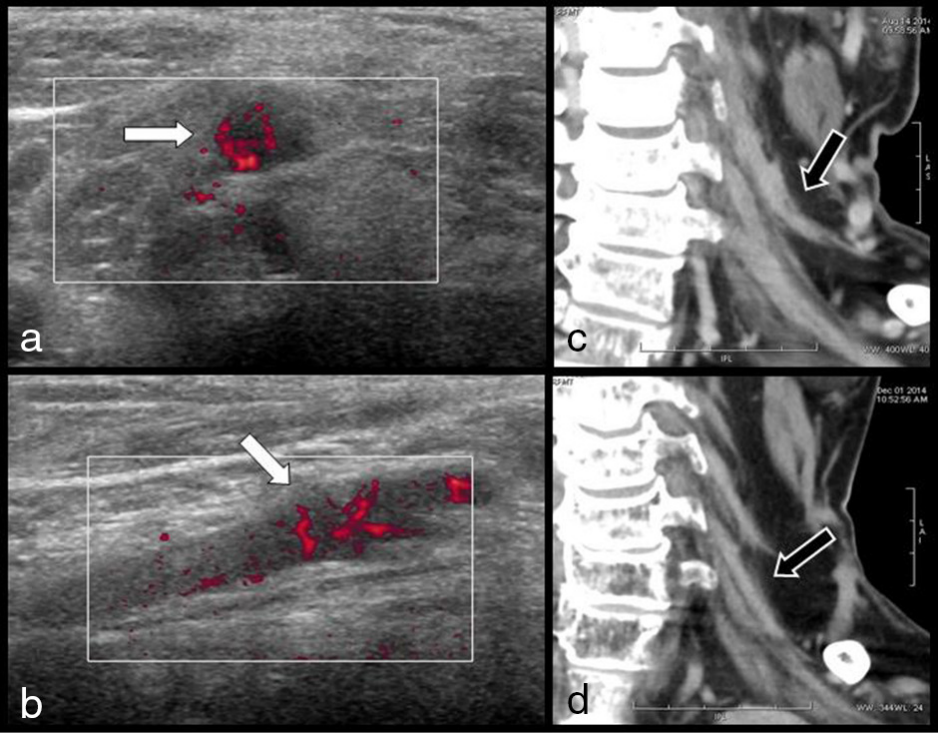
Transverse (a) and longitudinal (b) ultrasound views of the most swollen sixth root. The root is hypoechoic, thickened with irregular and hazy outlines (white arrow). Colour Doppler ultrasound reveals rich anarchic vascularization of the pathologic root. Coronal multidetector CT reconstructions before (c) and 3 months after (d) radiotherapy show a considerable decrease in the caliber of the roots (black arrow). Only some contrast enhancement persists.

Complementary MRI of the cervical spine confirmed the diagnosis of substantial cervical stenosis and surgical treatment was planned. However, in the meantime, electromyography revealed diffuse multiradicular alterations of the cervicobrachial plexus and complementary MRI (Figures 1 and 2a) of the BP was prescribed before surgery. *T*_1_ weighted images first showed diffuse atrophy of the scapular muscles with typical hyperintense denervation oedema on *T*_2_ weighted corresponding images. Diffuse thickening of the BP roots with high signal intensity (SI) on *T*_2_ weighted images was found to extend from the cervical spine to the axilla. *T*_2_ weighted images obtained more distally in the mediastinum revealed a tumoral mass in the inferolateral portion of the anterior mediastinum. Complementary cervicothoracic contrast-enhanced MDCT was performed to complete the staging of this mediastinal mass (Figure 1b–d). The CT scan confirmed diffuse, irregular, tortuous contrast-enhancing thickening of the third, fourth and fifth and especially of the sixth left cervicobrachial roots. This thickening extended from the neurovascular foramina to the axilla. The staging of the mediastinal tumour was continued with ^18^F-FDG PET/CT images, which also clearly illustrated the high FDG uptake within the thickened roots (Figure 1e,f). The diagnosis of multiradicular MP was retained and reinforced by complementary empiric ultrasound study of the BP (Figure 3a,b). The cervicobrachial roots, especially the sixth root, appeared thickened and hypoechoic with irregular and hazy outlines and colour Doppler ultrasound revealed a rich anarchic vascularization. The patient underwent biopsy of the mediastinal mass through a limited thoracotomy and the diagnosis of poorly differentiated large-cell carcinoma of the lung was confirmed.

## Differential diagnosis

Before the specific diagnosis of brachial MP, pain experienced by the patient is often attributed, as also reported in our case, to various entities comprising bursitis or tendinitis of the shoulder, cervical arthritis, cervical disc prolapse, fibromyalgia, etc. It is particularly the case when the notion of cancer being responsible for the pain is lacking, as reported in our case. As a consequence, the diagnosis can be delayed for months.^1^ Weakness generally develops later.

The differential diagnosis of MP in cancer patients nevertheless also includes epidural cord compression, neoplastic meningitis, primary plexus tumours, rarely paraneoplastic plexopathy, post-infectious plexopathy and toxicity from intra-arterial chemotherapy. In addition, in patients with a history of radiotherapy, radiation-induced plexopathy may also affect up to 5% of patients.^2^

## Treatment and outcome

The patient was treated with chemotherapy, combining cisplatin and pemetrexed. He received four courses of chemotherapy and maintenance therapy was pursued with pemetrexed. The painful plexopathy was specifically treated with radiotherapy. The patient received a dose of 10 Gy in three sessions with posterior oblique beam and an equivalent of 36 Gy in 2 Gy conventional fractionation. A slow but significant improvement in cervicobrachial pain was obtained after a few weeks. Follow-up cervical MDCT (Figure 3c,d) and ^18^F-FDG PET/CT (not illustrated) demonstrated a rather drastic regression of the radicular thickening. Cervicobrachial pain, nevertheless, reappeared gradually after 5 months and a second session of radiotherapy was considered but the patient died a few weeks later because of multisystem decompensation.

## Discussion

When compared with toxic, metabolic and even paraneoplastic involvement of peripheral nerves, true neoplastic lesions of the nerves roots are much rarer.^2^ The exact mechanism of neoplastic nerve and/or plexus involvement remains unclear and varies according to the type of the tumour.^2^ Empiric classification of these mechanisms is based on morphologic criteria. Infiltration may occur within the cerebrospinal fluid space, per continuitatem, by intravascular spread (specific spread of lymphoma), by retrograde infiltration (frequently described in tumours of the face, ear, nose and throat but also common in BP tumours) or through the dorsal root ganglia.^1^ Direct nerve metastases is a rare occurrence. On the contrary, direct nerve infiltration from adjacent organs is more common and occurs *via* lymph nodes in the BP and sacral plexus or by direct infiltration into the BP in lung cancer.^1,3^ Direct mechanical compression, circumferential cuffing or compression owing to dural infiltration are other mechanisms.^2^

There is a theoretical anatomic distinction between the cervical plexus (upper four cervical roots) and the BP (lower four cervical and first dorsal roots + occasionally the C4 and T2 roots). These two adjacent plexus are the sites of local metastasis or infiltration from adjacent tumours such as lung cancer, breast cancer and also lymphoma. Melanoma, sarcoma, squamous cell carcinoma arising in the head and neck, and malignant mesothelioma are less frequent.^2–5^

It is hypothesized that tumours spread mainly by lymphatic spread.^4^ Due to the vicinity of the lymphatics of the apical lung lower BP lesions are much more frequent than upper BP lesions.^2,4^ In addition to direct compression and infiltration, centripetal tumour can spread *via* the nerve roots and by direct extension into the epidural space can cause root infiltration or epidural spinal cord compression.^2^

In breast carcinoma, plexus infiltration is generally a rather late complication. The infiltration usually occurs *via* the lymphatics related to the lateral group of axillary lymph nodes. Centripetal tumoral spread along the nerve roots may also produce with secondary invasion of the epidural space and finally spinal cord compression. In lymphoma, BP infiltration is generally a late complication. Upper BP infiltration is more frequent. In 10–15% of cases, Hodgkin disease may affect the BP.

Lung cancer develops in the apical area of the lung in about 5% of non-small-cell lung cancer and 1% of small-cell lung cancer cases.^6^ Apical lung tumour (Pancoast syndrome) can directly spread to the C8 and T1 nerve roots or to the BP (inferior trunk, C8–T1; and medial cord, C7). Horner’s syndrome,^4^ not described in our patient, is a common finding in brachial MP related to Pancoast syndrome. Lung cancer metastasis to the axillary and/or supraclavicular nodes can also secondarily involve the BP.^4,6^ In the reported case, the primary tumour was situated in the left anterior and superior mediastinum at a distance from the apical lung but in an area likely to drain into the left supraclavicular area.

Invasion of the BP only results in pain (75% of cases), followed by numbness and dysethesias (25% of cases).^1,4^ The pain classically irradiates through the shoulder, axilla, medial arm and forearm into the fourth and fifth fingers.

The diagnosis of malignant nerve or plexus infiltration or metastases is case and tumour dependent and lacks of any standardized diagnostic scheme.^2^ For infiltration of nerves or plexus, clinical examination and electrophysiology are unspecific. The definite diagnosis depends heavily and exclusively on medical imaging. MRI has been claimed to be the optimal imaging modality for investigating the BP. It is superior to CT scan thanks to its multiplanar capabilities—an advantage that appears actually less critical now owing to the development of MDCT—and because it differentiates more accurately the nerves among surrounding vessels and soft tissues. MRI has better contrast resolution for soft tissues than for CT scan.^7^ MRI can detect metastatic infiltration or spread of cancer along nerves.^2,6,7^ Roots of the BP show low SI on *T*_1 _ and intermediate/slightly high SI on *T*_2_ weighted images. The adjacent fat, through which the nerves travel, provides superior intrinsic contrast on *T*_1_ weighted images.^7^ Typically, metastases of the BP have a low SI on *T*_1_ and a high SI on *T*_2_ weighted images.^5^ Nevertheless, the lesions may also occasionally show low SI on *T*_2_ weighted images. Metastases classically show enhancement on *T*_1_ weighted fat-saturated gadolinium-enhanced images. In lymphoma, enlarged nodes can infiltrate the plexus or directly compress it. Neurolymphomatosis is a rare expression of lymphoma involving the peripheral nerves, which can also invade the BP. MRI shows diffuse thickening of the plexus roots with marked hyperintensity on *T*_2_ weighted images and contrast enhancement.

In patients having previously received radiation therapy, for example in breast cancer, post-radiation plexopathy and radiation fibrosis are not always easy to differentiate from MP because of an overlap of the clinical and imaging features. Radiation plexopathy often presents with oedema-related sensory symptoms. On the contrary, pain dominates the clinical presentation of MP and can be associated with Horner's syndrome.^7^ Nevertheless, there are many MRI similarities between MP and radiation plexopathy. Fibrosis is less ambiguous, showing low intensity on both *T*_1_ and *T*_2_ weighted images. *T*_2_ fat-suppressed and short tau inversion-recovery sequences help to distinguish fibrosis from cancer recurrence.^5^

^18^F-FDG PET/CT combined with CT or MRI is currently recommended for evaluation of patients with suspect MP, especially in ambiguous cases, or when other imaging techniques are doubtful or appear normal.^3,6^ ^18^F-FDG PET/CT is particularly recommended in patients presenting with breast carcinoma not only because it can evaluate treatment efficacy but also when there is a suspicion of infiltration of the BP.^2,8^ ^18^F-FDG PET/CT is superior to MRI for distinguishing real tumoral involvement from secondary radiation plexopathy. ^18^F-FDG PET/CT also has good sensibility for the diagnosis of neurolymphomatosis.

Recent literature is lacking concerning the new performances of MDCT in the study of BP diseases. Our experience is also insufficient to make any conclusions. Nevertheless, in our case, diffuse irregular thickening of the metastatic plexus roots with massive contrast enhancement was highly suggestive. Moreover, thanks to its high performance in multiplanar reconstructions, MDCT allows high quality images of the pathologic BP that are equivalent to MRI.

In the reported case, ultrasound of the BP was additionally performed. It not only proved feasible but also appeared to be an interesting diagnostic method to explore MP. An irregular hypoechoic fusiform thickening of the roots was demonstrated.^9^ Moreover, the thickened roots showed very hazy outlines and colour Doppler also demonstrated an anarchic tumoral neovascularization. Ultrasound of the BP is already commonly used by anaesthetists for ultrasound-guided BP blocks. Nevertheless, its use should be encouraged for other indications comprising thoracic outlet syndrome, BP trauma, MP and radioinduced fibrosis, neurogenic tumours and Parsonage–Turner syndrome.^7,10^

## Conclusions

The reported case typically illustrates that several imaging modalities today are able to diagnose brachial MP. If MRI remains the gold standard for imaging this area, we also illustrated the capabilities of contrast-enhanced MDCT and ultrasound with colour Doppler. Moreover, we also confirmed the specificity of FDG–PET to distinguish brachial MP from, for example, secondary radiation plexopathy. As a consequence, an open surgical exploration with biopsies of brachial MP, which carries the risk of permanent deficits, is now rarely necessary.^2^

## Learning points

1. Brachial MP is a rare event, often resulting in severe pain and muscular weakness. Lung cancer, breast cancer and also lymphoma are the most common concerned neoplasms.
2. MRI represents the optimal gold standard imaging modality to investigate the BP.
3. ^18^F-FDG PET/CT is highly recommended for patients with suspect MP, especially in ambiguous cases or when other imaging techniques are doubtful or appear normal. ^18^F-FDG PET/CT is more specific than MRI in distinguishing MP from secondary radiation plexopathy.
4. Ultrasound study of the BP is feasible and should be encouraged as a complementary or alternative method to MRI. MDCT could also represent an alternative or complementary imaging method thanks to its high performance in multiplanar reconstructions

## Consent

The patient died 6 months ago and a written consent was obtained from the family. Moreover, images have been completely anonymized and this point has been scrupulously checked.
